# Carbon-Based Nanomaterials

**DOI:** 10.3390/ijms22147726

**Published:** 2021-07-20

**Authors:** Ana María Díez-Pascual

**Affiliations:** Universidad de Alcalá, Facultad de Ciencias, Departamento de Química Analítica, Química Física e Ingeniería Química, Ctra. Madrid-Barcelona, Km. 33.6, 28805 Alcalá de Henares, Madrid, España (Spain); am.diez@uah.es; Tel.: +34-918-856-430

Research on carbon-based nanomaterials, such as carbon nanotubes, graphene and its derivatives, nanodiamonds, fullerenes, and other nanosized carbon allotropes, has experienced sharp exponential growth over recent years. The infinite possibilities to modify and tailor carbon nanomaterials are associated with their small size, approaching the size of many fundamental biomolecules, their large specific surface area, high electrical and thermal conductivity, unique optical properties, and superior mechanical properties, which have paved the way for a broad range of applications. In particular, fullerene derivatives have been applied to solar energy scavenging, graphene has been widely used in flexible electronics, carbon nanotubes have been tailored to have molecular recognition capability, graphene quantum dots have been extensively used for bio-imaging and sensing owing to their photoluminescence properties, and nanodiamonds have been demonstrated to be useful in super-resolution imaging and nanoscale temperature sensing.

This Special Issue “Carbon-Based Nanomaterials” (https://www.mdpi.com/journal/ijms/special_issues/carbon-ijms (accessed on 30 September 2020)) and Special Issue “Carbon-Based Nanomaterials 2.0” (https://www.mdpi.com/journal/ijms/special_issues/carbon_nano_2 (accessed on 30 September 2020)) with a collection of 13 original contributions and 5 literature reviews, provides selected examples on surface modifications of carbon nanomaterials to tailor their physicochemical properties as well as their applications in a variety of fields, such as electronics, energy storage, biomedicine, and sensing.

The last two decades have witnessed a lot of research addressing the possible biomedical applications of carbon nanotubes (CNTs) such as drug delivery, tissue engineering, diagnostics, and biosensing [1]. In particular, CNTs can act as contrast agents in different imaging methods [2]. Upon functionalization and conjugation with various biomarkers, they can indicate the presence and localization of targeted cells with good spatial resolution. CNTs are suitable candidates to be employed in solving the theragnostic challenges associated with neurological diseases such as ischemic stroke. In this regard, Komane et al. [3] recently synthesized vertically aligned multiwalled carbon nanotubes (VA-MWCNTs), which were purified, carboxylated, acylated, and PEGylated, for use in targeting studies. The functionalized VA-MWCNTs were found to be nontoxic towards PC-12 neuronal cells, a type of catecholamine cell that synthesizes, stores, and releases norepinephrine and dopamine.

Nerve regeneration via cell electrostimulation will turn out to be essential in regenerative medicine, particularly in body reconstruction, artificial organs, or nerve prostheses. Indirect electrical cell stimulation needs a nontoxic, highly electrically conductive substrate material allowing for an accurate and effective cell electrostimulation. This can be effectively achieved using graphene nanoplatelets (GNPs); however, their strong agglomerating tendency hinders the quality of the manufactured coatings. Therefore, the choice of an appropriate amount of surfactant to achieve both a high conductivity and quality of the coating is crucial. In this regard, Dybowska-Sarapuk et al. [4] developed graphene inks with different surfactant contents in the range of 2–20 wt% and demonstrated an observable effect of electrostimulation on the behavior of the neuronal stem cells embedded in the graphene layer. The use of cellular electrostimulation may provide a solution to the currently irredeemable neurological disorders, e.g., possibilities for the restoration of the spinal cord and nerve connections.

On the other hand, biocompatible and water-soluble C_60_ fullerenes can inactivate free radicals, including methyl radicals, superoxide anion radicals, and hydroxyl radicals, protecting cell membranes from oxidation. They are powerful scavengers of free radicals during the development of ischemia and fatigue processes in skeletal muscle [5]. The usage of safe doses of C_60_ fullerene at the initiation of various pathologies leads to significant positive therapeutic effects, in particular, during acute liver injury, colorectal cancer, obesity, acute cholangitis, and hemiparkinsonism and can be effective nanotherapeutics in the treatment of certain herbicide poisoning such as glyphosate [6].

Another interesting biomedical application of carbon nanomaterials is in vitro bio-imaging. In this regard, Parasuraman et al. [7] synthesized “dahlia-like” hydrophilic fluorescent carbon nanohorns (CNH) via a simple hydrothermal chemical oxidation method from Nafion-encapsulated carbon nanorice particles at a mild temperature of 100 °C. The CNHs obtained could be used as bio-imaging probes because of the presence of structural defects such as 5-hydroxymethylfurfural or other aromatic moieties generated during the carbon nanorice oxidation. The synthesis method developed in this study will pave a new way for the application of CNHs as bio-imaging agents and drug carriers.

Graphene and its derivatives are very promising nanomaterials for the preparation of scaffolds for tissue repair. The response of immune cells to these graphene-based materials appears to be critical in promoting regeneration; thus, the study of this response is essential before they are used to prepare any type of scaffold. Another relevant factor is the variability of graphene-based materials, including the surface chemistry, namely, the type and quantity of oxygen functional groups. Thus, Cicuéndez et al. [8] investigated the response of RAW-264.7 macrophages, monocyte-like cells, originating from an Abelson leukemia virus-transformed cell line derived from BALB/c mice, to graphene oxide (GO) and two types of reduced GO, rGO15 and rGO30, obtained after vacuum-assisted thermal treatment of 15 and 30 min, respectively. The results demonstrate that the GO reduction led to a decrease in both oxidative stress and proinflammatory cytokine secretion, considerably enhancing its biocompatibility and potential for the preparation of novel 3D scaffolds able to trigger the immune response for tissue regeneration.

Another approach is to take advantage of the chemically inert properties of graphene for coating medical devices. For effective coating, it is necessary to prepare the surface of the substrate to be coated and to optimize the chemical vapor deposition process, allowing better attachment, e.g., by using the defective interface side of the graphene layer for bonding. In this regard, Wasyluk et al. [9] synthesized a graphene coating on a Co-Cr alloy by a cold wall chemical vapor deposition (CW-CVD) method, with good mechanical properties (namely, hardness and elastic modulus). The results of the hemocompatibility test indicate that the developed coating does not have a pro-coagulant effect, thus corroborating its potential for medical applications, particularly in the field of cardiovascular diseases.

Graphene and its derivatives can also be used in anticancer therapy. For instance, they exhibit antitumor effects on glioblastoma multiforme cells in vitro. In this regard, the antitumor activity of rGO with different contents of oxygen-containing functional groups and graphene has been compared [10]. Cell membrane damage, changes in the cell membrane potential, the gene expression of voltage-dependent ion channels, and extracellular receptors were analyzed. A reduction in the potential of the U87 glioma cell membrane was observed after treatment with rGO/ammonium thiosulphate and rGO/thiourea dioxide flakes. Treatment with graphene or rGO led to reduced endoglin expression, stimulated cell adhesion, and, hence, reduced the ability of cancer cell migration.

rGO has also emerged as a good candidate for cancer photothermal therapy due to its huge specific surface area for drug loading, high biocompatibility, targeted delivery, outstanding photothermal conversion in the near-infrared range, and functional groups for functionalization with molecules such as photosensitizers, siRNA, and ligands [11]. Multifunctional rGO-based nanosystems can be designed, which possess promising temperature/pH-dependent drug/gene delivery abilities for multimodal cancer therapy.

On the other hand, pharmaceuticals are recently being considered emergent micropollutants due to their potential environmental, ecotoxicological, and sociological risks. The anaerobic removal of pharmaceuticals assisted by nanomaterials constitutes a promising strategy that is worthy of investigation. In this regard, Silva et al. [12] evaluated the potential of commercial and custom-made CNTs as redox mediators in the anaerobic removal of ciprofloxacin, a fluoroquinolone antibiotic widely used for the treatment of bacterial infections in humans and animals. Functionalized CNTs with different surface chemical groups (acidic and basic), and magnetic CNTs incorporating iron were prepared and used in biological experiments, since they are easier to recover and may be reused, which is important for applied biological treatment processes. The potential contribution of adsorption and biodegradation processes was assessed. The amount of carbon nanomaterial needed was found to be very low, only 0.1 g L^−1^, which minimizes the quantity of the carbon nanomaterial that can be released to the medium.

Clean energy technologies constitute another hot topic of research in the field of carbon nanomaterials. Hydrogen fuel, a valuable alternative to fossil fuels, shows drawbacks such as the poisoning by impurities of the metal catalyst controlling the reaction involved in its production. Thus, separating H_2_ from other gases such as CO, CO_2_, CH_4_, N_2_, and H_2_O is essential. Muraru and Ionita [13] presented a rotating partially double-walled CNT membrane designed for hydrogen separation and assessed its performance using molecular dynamics simulations by imposing three discrete angular velocities. They demonstrated that the angular velocity plays a noteworthy role in hydrogen separation through a rotating nanotube.

Owing to both the extremely high in-plane/shell strength and modulus and extremely low inter-plane/shell friction, CNTs and graphene are widespread in developing nanodevices, e.g., oscillators, nanomotors, nanobearing, and nanoresonators. Generally, MWCNTs are used in experiments or simulations for investigating the inter-tube friction. In particular, it was found that an abrupt jump in the output torque moment from a rotation transmission nanosystem made from CNTs occurred when reducing the system temperature. When the rotor was subjected to an external resistant torque moment, it could not rotate opposite to the motor, even if it deformed severely. Combining molecular dynamics simulations with the bi-sectioning algorithm, the critical value of the resistant torque moment was determined [14].

Graphene and its derivatives are also promising materials for the development of a new generation of gas separation membranes, mainly because of their thinness, excellent membrane formation capabilities, permeability, and selectivity. In particular, GO-based membranes can allow extremely high fluxes because of their fineness and layered structure. In addition, their high selectivity is due to the molecular sieving or diffusion effect resulting from their narrow pore size distribution and unique surface chemistry. In this regard, the different mechanisms of gas transport and gas separation by GO membranes, as well as the methods for GO membrane preparation and characterization, have been reviewed [15].

Fullerenols, nanosized water-soluble polyhydroxylated derivatives of fullerenes, are bioactive compounds that can be used for drug development. Kovel et al. [16] investigated the antioxidant activity and toxicity of a series of fullerenols with a different number of oxygen substituents. All fullerenols caused toxic effects at high concentrations (>0.01 g L^−1^), whereas their antioxidant activity was demonstrated at low and ultralow concentrations (<0.001 g L^−1^). The toxic and antioxidant characteristics of the fullerenols were found to be dependent on the number of oxygen substituents: the fewer the oxygen substituents, the lower the toxicity, and the better the antioxidant activity. The differences in fullerenol properties were ascribed to their catalytic activity due to the reversible electron acceptance, radical trapping, and balance of reactive oxygen species in aqueous solutions. The results provide the basis for the selection of carbon nanomaterials with appropriate toxic and antioxidant characteristics.

On the other hand, the use of carbon nanomaterials as antibacterial agents has been widely investigated. It constitutes a very promising way to fight microorganisms due to their high specific surface area and intrinsic or chemically incorporated antibacterial action. In particular, graphene and its derivatives, GO and rGO, are highly suitable candidates for restricting microbial infections. However, the mechanisms of antimicrobial action, their cytotoxicity, and other issues remain unclear. Selected examples on the preparation of antimicrobial nanocomposites incorporating inorganic nanoparticles and graphene or its derivatives have been provided [17]. The antibacterial property of composites of rGO with Ag and ZnO nanoparticles synthesized using a microwave-assisted approach has been investigated in detail [18]. A strong antibacterial activity against *E. coli* and *S. aureus* was found since they decreased the reductase activity and affected the membrane integrity in the bacteria. These nanocomposites can be applied not only as antibacterial agents but also in a variety of biomedical materials such as sensors, photothermal therapy, drug delivery, and catalysis.

Over recent years, diverse nanomaterials have been investigated to design highly selective and sensitive sensors, reaching nano/picomolar concentrations of biomolecules, which is crucial for medical sciences and the healthcare industry in order to assess physiological and metabolic parameters. In particular, G and its derivatives are becoming crucial in the field of optical and electrochemical sensors. G-based nanomaterials can be combined with inorganic nanoparticles, including metals and metal oxides, quantum dots, organic polymers, and biomolecules, to yield a wide range of nanocomposites with enhanced sensitivity for sensor applications. Recent research on G-based nanocomposites for the detection of bioactive compounds, including melatonin, gallic acid, tannic acid, resveratrol, oleuropein, hydroxytyrosol, tocopherol, ascorbic acid, and curcumin, has been reviewed [19], and the sensitivity and selectivity of G-based electrochemical and fluorescent sensors have been analyzed. Some of these bioactive compounds, such as tannic acid, can be used as effective dispersing agents for graphene in aqueous solutions [20], and tannic acid’s interaction with a water-soluble vitamin, riboflavin, has been studied under different experimental conditions. Tannic acid induces quenching of riboflavin fluorescence, and the effect is stronger with an increasing tannic acid concentration, due to π–π interactions through the aromatic rings, and hydrogen bonding interactions between the hydroxyl moieties of both compounds.

Finally, the recent advances in the synthesis of mesoporous carbon, with a high surface area, large pore volume, and good thermostability, make it a promising material with a wide range of applications. It can act as a catalytic support, can be used in energy storage devices, controls the body’s oral drug delivery system, and adsorbs poisonous metals from water and various other molecules from aqueous solutions. The potential applications of mesoporous carbon in many scientific disciplines have been reviewed [21]. Particular emphasis has been placed on some areas, especially surface modification, oxidation, and functionalization time for certain applications. The role of the reaction pH, reaction time, time of carbon surface oxidation, functionalization parameters, solvent uses during adsorption, and optimal time in adsorption has been discussed. Moreover, the outlook for further improvement in mesoporous carbon has been demonstrated.
